# An Approach to Maximize Retrograde Transport Based on the Spatial Distribution of Motor Endplates in Mouse Hindlimb Muscles

**DOI:** 10.3389/fncel.2021.707982

**Published:** 2021-08-11

**Authors:** Jianyi Xu, Ang Xuan, Zhang Liu, Yusha Li, Jingtan Zhu, Yingtao Yao, Tingting Yu, Dan Zhu

**Affiliations:** ^1^Britton Chance Center for Biomedical Photonics, Wuhan National Laboratory for Optoelectronics, Huazhong University of Science and Technology, Wuhan, China; ^2^MoE Key Laboratory for Biomedical Photonics, School of Engineering Sciences, Huazhong University of Science and Technology, Wuhan, China

**Keywords:** motor endplate, spatial distribution, 3D intramuscular injection, retrograde transport, tissue optical clearing

## Abstract

Knowledge regarding the relationship between muscles and the corresponding motor neurons would allow therapeutic genes to transport into specific spinal cord segments. Retrograde tracing technique by targeting the motor endplate (MEP), a highly specialized structure that offers direct access to the spinal motor neurons, has been used to elucidate the connectivity between skeletal muscles and the innervating motor neuron pools. However, current injection strategies mainly based on blind injection or the local MEP region might lead to an underestimation of the motor neuron number due to the uneven distribution of MEP in skeletal muscles. In this work, we proposed a novel intramuscular injection strategy based on the 3D distribution of the MEPs in skeletal muscles, applied the 3D intramuscular injection to the gastrocnemius and tibialis anterior for retrograde tracing of the corresponding motor neurons, and compared this with the existing injection strategy. The intramuscular diffusion of the tracer demonstrated that 3D injection could maximize the retrograde transport by ensuring a greater uptake of the tracer by the MEP region. In combination with optical clearing and imaging, we performed 3D mapping and quantification of the labeled motor neurons and confirmed that 3D injection could label more motor neurons than the current injection method. It is expected that 3D intramuscular injection strategy will help elucidate the connective relationship between muscles and motor neurons faithfully and becomes a promising tool in the development of gene therapy strategies for motor neuron diseases.

## Introduction

Dysfunctions or diseases of the lower motor neurons are among the most debilitating motor disorders and influence the life quality of the affected individuals (Mcdonald and Sadowsky, 2002; Anderson, 2004; Gupta et al., 2010). Traumatic spinal cord injury causes irreversible damage to the motor, sensory, and/or autonomic systems, leading to partial or complete paralysis and even death. Some neurodegenerative diseases can also result in a substantial loss of spinal cord function, such as amyotrophic lateral sclerosis, spinal muscular atrophy, and Duchenne muscular dystrophy. Until now, there is still less effective treatment to cure the spinal cord and restore function in clinical practice (Silva et al., 2014).

Recent development in gene transfer techniques has provided a new strategy to treat spinal cord injury and diseases (Walthers and Seidlits, 2015). The therapeutic transgenes can be transferred to motor neurons *via* direct injection (Lepore et al., 2007; Franz et al., 2009; Klaw et al., 2013), systemic injection (Duque et al., 2009; Snyder et al., 2011; Hordeaux et al., 2015), or intramuscular injection (Boulis et al., 2003; Towne et al., 2010; Tosolini and Morris, 2016; Hardcastle et al., 2018) to the spinal cord. Thereinto, intramuscular injection is particularly promising because it is easily accessible and less invasive and has been recently used in animal models of motoneuron diseases (Wang et al., 2002; Benkhelifaziyyat et al., 2013). *Via* intramuscular injection, the therapeutic gene vectors are transported retrogradely to spinal motor neurons connected to the injected muscles. Thus, accurate knowledge regarding the anatomical relationship between spinal motor neurons and the muscles that they innervate is necessary for specific and efficient transduction of therapeutic vectors.

Retrograde tracing techniques have been widely applied to define the connectivity between skeletal muscles and the innervating motor neuron pools (Richmond et al., 1994; Haenggeli and Kato, 2002; Hayashi et al., 2007; Lanciego and Wouterlood, 2011; Vandeweerd et al., 2018). And motor endplate (MEP), as a highly specialized structure in muscles, offers direct access for retrograde tracers to the presynaptic terminals and then to spinal motor neurons. However, the MEPs are not evenly distributed in skeletal muscles (Nandedkar et al., 1988; Prodanov et al., 2005). The accuracy of a tracer injection targeting the MEPs determines the labeling efficiency and the interpretation of the relationship between spinal motor neurons and muscles (Shaari and Sanders, 1993; Chin et al., 2005; Gracies et al., 2009; Van Campenhout and Molenaers, 2011). Tosolini and Morris (2012), Tosolini et al. (2013), and Mohan et al. (2014, 2015b) characterized the position of the MEP region on the muscle surface using acetylcholinesterase histochemistry, providing a map and guide for intramuscular tracer injection. This injection strategy based on the MEP location on the muscle surface was still blind to a certain extent due to the lack of a spatial distribution of MEPs in the intact muscles. More recently, Yin et al. (2019) obtained the spatial distributions of the MEPs in whole skeletal muscle based on tissue optical clearing techniques. They provided a potential 3D map for the intramuscular injection of dyes or tracers.

Here we propose a novel injection strategy based on the 3D distribution of MEPs and define it as “3D intramuscular injection.” We labeled the spinal motor neurons corresponding to the gastrocnemius and tibialis anterior *via* intramuscular injections of retrograde tracers based on the reproduced spatial and surficial MEP distributions, respectively. To identify the labeling efficiency, we compared the tracer diffusion and the labeled spinal motor neurons of two injection strategies qualitatively and quantitatively. The newly proposed 3D injection strategy provides a valuable guide for intramuscular injection in future scientific researches and clinical treatment. This work is also expected to provide a framework for the exploration of the muscle–motoneurons topographical relationship.

## Materials and Methods

### Animals

All animal care and all experimental protocols were conducted according to the Experimental Animal Management Ordinance of Hubei Province, P.R. China, and have been approved by the Institutional Animal Ethics Committee of Huazhong University of Science and Technology. C57BL/6J mice (7–8 weeks old) weighing 20 ± 2 g were purchased from Liaoning Changsheng Biotechnology Co., Ltd., and were group-housed with their littermates in a dedicated housing room under a 12-h light/12-h dark cycle, and food and water were available *ad libitum*.

### Retrograde Tracers

As retrograde tracers used extensively, AlexaFluor 647-conjugated cholera toxin subunit B (CTB) (CTB-647, Invitrogen, Carlsbad, CA, United States) was diluted in 0.01 M phosphate-buffered saline (PBS, Sigma-Aldrich, St. Louis, MO, United States) at a concentration of 1 μg/μl (0.1%) and then used for intramuscular injections. The solutions were stored at −20°C until use.

### Labeling of the MEPs

To label all MEPs in whole muscles, an *in vivo* injection of a fluorescent tracer was conducted according to the published literatures (Chen et al., 2016; Yin et al., 2019). AlexaFluor 647-conjugated α-bungarotoxin (α-BTX 647, Invitrogen, Carlsbad, CA, United States) or AlexaFluor 594-conjugated α-bungarotoxin (α-BTX 594, Invitrogen, Carlsbad, CA, United States) was injected *via* the tail vein at a dose of 0.3 μg/g, with a 2-h conjugation time before perfusion.

To display the MEPs on the muscle surface, acetylcholinesterase histochemistry staining was performed by referring to the protocol (Mohan et al., 2015a). The mixture solution was prepared by adding 72.5 mg acetylthiocholine iodide (Aladdin, Shanghai, China), 150.0 mg glycine (Aladdin, Shanghai, China), and 105.0 mg copper sulfate (Aladdin, Shanghai, China) into 50 ml of 0.1 M phosphate buffer in turns until all products are completely dissolved. After perfusion, the entire mouse hindlimb was dissected, and the skin and fascia were removed to ensure the exposure of the muscles to the mixture solution. Then, the whole hindlimb was immersed into the mixture solution overnight at 4°C. After immersion, the hindlimb was washed with distilled water (dH_2_O) three times and subsequently incubated in 5% ammonium sulfide (Aladdin, Shanghai, China) solution for a few seconds until the muscles turned brown. Finally, the hindlimb was washed with dH_2_O and was stored in 0.01 M PBS.

### The Procedure of Intramuscular Injections

Anesthesia was induced with a mixture of 2% α-chloralose and 10% urethane (8 ml/kg) through an intraperitoneal injection. The fur covering the targeted hindlimb was shaved and cleaned firstly with 75% ethanol. Then, the gastrocnemius was exposed by making an incision between the popliteal fossa and the ankle of the lower hindlimb. The tibialis anterior was exposed by making an incision on the anterolateral calf of the lower hindlimb. CTB-647 was diluted to 1 μg/μl (0.1%) with 0.01 M PBS and injected into the muscles using a glass micropipette (Drummond Scientific Company, Broomall, PA, United States) connected to a syringe pump (Drummond Scientific Company, Broomall, PA, United States). To ensure accuracy and repeatability, a stereotaxic instrument (RWD Life Science Co., Ltd., Shenzhen, China) was used to quantify the depth of each injection. Special care was taken to ensure that the blood vessels around the muscles were intact and the fascia surrounding the injection sites was minimally disrupted. CTB-647 was slowly injected into the muscles at a rate of 3 nl/s, with the micropipette being held in place for an additional 5 min after injection before being slowly retracted from the muscles. After injection, the muscles were wiped with gauze to clear any tracer that may unexpectedly seep from the injected site. The incision was sutured with 5-0 nylon sutures. To provide pain relief, 4% w/v tolfedine (Vetoquinol, France, 0.1 ml/20 g) was given by intraperitoneal injection half an hour before the surgery and for three postoperative days.

### Tissue Harvesting

After the intramuscular injections, the mice were kept for 3 days to allow the optimal retrograde transport of CTB (Hirakawa et al., 1992). Then, the mice were anesthetized deeply and intracardially perfused with 0.01 M PBS followed by 4% paraformaldehyde (PFA, Sigma-Aldrich, St. Louis, MO, United States) in 0.01 M PBS. After perfusion, an incision was made on the ventral aspect to remove the viscera and the posterior abdominal wall muscles. The entire spinal cord segment was dissected out from the T12 nerve root to the L2 nerve root to make sure that the labeled motor neuron pool was covered. The spinal nerve roots were preserved to contribute to recognizing the segments of the spinal cord. Then, the spinal cord segment was attached with 5-0 sutures to a piece of folded aluminum foil to be kept flat and was post-fixed overnight in 4% PFA at 4°C.

The gastrocnemius, tibialis anterior, soleus (SOL), and extensor digitorum longus (EDL) were dissected out and post-fixed overnight in 4% PFA at 4°C. Then, the muscles were rinsed with 0.01 M PBS several times. To investigate the diffusion of CTB in skeletal muscle and its coverage on the MEP region, the muscles were embedded in 4% agarose and sliced into 400 μm (for the gastrocnemius and soleus) and 300 μm (for the tibialis anterior and EDL) longitudinal sections with a vibratome (Leica VT1000, Wentzler, Germany), respectively.

### Optical Clearing Procedure

The FDISCO protocol was performed according to the original literature (Qi et al., 2019). Before clearing, tetrahydrofuran (THF, Sigma-Aldrich, St. Louis, MO, United States) and dibenzyl ether (DBE, Sigma-Aldrich, St. Louis, MO, United States) were preprocessed by column absorption chromatography with basic activated aluminum oxide (Sinopharm Chemical Reagent Co., Ltd., Shanghai, China) to remove the peroxides. The spinal cords were dehydrated in THF solutions diluted in dH_2_O (pH adjusted to 9.0−9.5 with trimethylamine; Sinopharm Chemical Reagent Co., Ltd., Shanghai, China) at graded concentrations as follows: 50, 70, 80, and 100 vol% (twice) for 45−60 min at each step. After dehydration, pure DBE was used as a refractive index matching solution to clear the spinal cord. During the clearing process, the spinal cord was placed in a glass chamber under the dark. All steps were performed at 4°C with slight shaking.

### Imaging

To obtain the spatial distribution of the MEPs in skeletal muscles, the cleared muscles were imaged using a light sheet fluorescence microscope (LaVision BioTec I, Bielefeld, Germany) equipped with an sCMOS camera (Andor Neo), a ×2/0.5 objective lens equipped with a dipping cap, and an Olympus MVX10 zoom microscope body (magnification range of ×0.63–×6.3). The cleared tissues were mounted on the sample holder and incubated with DBE in the sample reservoir. The z-step interval was 5 μm.

To analyze the labeled motor neurons or the intramuscular diffusion of the CTB, the whole spinal cord or muscle slices were imaged with inverted confocal fluorescence microscopy (LSM710; Zeiss, Oberkochen, Germany) equipped with Fluar ×5/0.25 objective (dry, W.D. 12.5 mm) and Fluar ×10/0.5 objective (dry, W.D. 2.0 mm). The cleared spinal cord segments or muscle slices were placed on a coverslip with the clearing reagent (DBE) or 0.01 M PBS, with another coverslip being put on the top of the sample.

### Image Processing and Analysis

The obtained images were processed and analyzed by ImageJ (NIH, Bethesda, MD, United States), Imaris (Bitplane, Zurich, Switzerland), and MATLAB (Mathworks^TM^, Natick, MA, United States). The maximum intensity projection of the z stack was performed with sequential images in ImageJ. The 3D visualization of the MEP distribution in the whole skeletal muscles and spinal motor neurons was performed in Imaris, and the distribution characteristics of the labeled motor neurons were analyzed quantitatively *via* the Spot and Vantage modules in Imaris. A custom-written code implemented in Matlab was used to calculate the coverage between the intramuscular tracer and the MEP distribution.

### Statistical Analysis

The SPSS (IBM Corp., Armonk, NY, United States) was used for statistical analysis in this work. The data are presented as mean ± SEM. For the analysis of statistical significance, the data distribution in each experiment was checked for normality using the Shapiro–Wilk test. The heterogeneity of variance was checked by the Levene test. *p*-values were calculated using the independent-sample *t*-test (two-sided) to compare the data between two groups (Figures 3G–I, 5D–F). If the data distribution is non-normal, the non-parametric test (Mann–Whitney *U*-test) was used (Figure 3G). If the data distribution is normal and the variance is not homogeneous, the separate variance estimation *t*-test was used (Figure 3G).

## Results

### A New Injection Strategy for Maximizing Retrograde Transport

Motor endplates offer a direct access to the presynaptic nerve terminals; hence, injecting tracers specifically into the entire MEP region would allow an efficient uptake of the tracers by the presynaptic nerve terminals and maximize the retrograde transport to achieve high-efficiency labeling of spinal motor neurons. The injection strategy proposed by Morris et al. that targets the MEPs on the muscle surface can improve retrograde transport but is still blind to a certain extent (Tosolini et al., 2013; Mohan et al., 2014).

**FIGURE 1:**
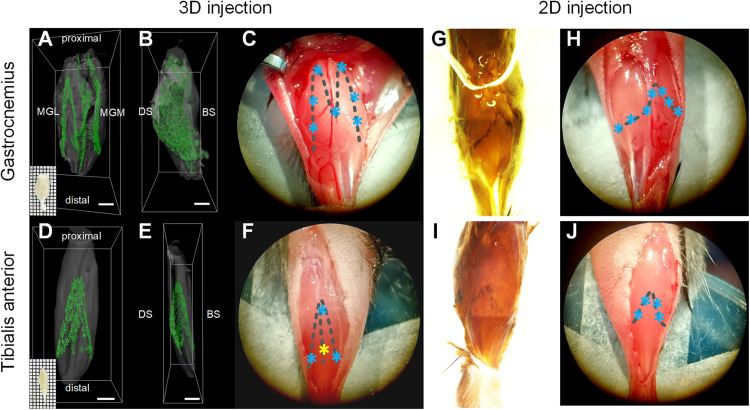
Illustration of intramuscular injection strategies based on the motor endplate (MEP) distributions. **(A–F)** The location of injection sites based on the 3D distributions of MEPs in the gastrocnemius and tibialis anterior. Panels **(A,B)** and **(D,E)** show the 3D distribution of MEPs of the gastrocnemius and tibialis anterior, respectively. Panels **(C,F)** show the injection sites for the 3D intramuscular injection of the gastrocnemius and tibialis anterior. The asterisks represent the injection sites. Note that the injection depth of the yellow site on the tibialis anterior is different from the other three sites. MGL, gastrocnemius lateralis; MGM, gastrocnemius medialis; DS, dorsal surface; BS, bone surface. Scale bar: 1,000 μm. **(G–J)** The location of injection sites according to the MEP regions on the muscle surface. Panels **(G,I)** show the MEP regions on the surfaces of the gastrocnemius and tibialis anterior following an acetylcholinesterase histochemical reaction. The injection sites along the surficial MEPs are shown in panels **(H,J)**.

In this study, we propose a new injection strategy based on the spatial distribution of MEPs in intact skeletal muscles, termed as “3D intramuscular injection.” To locate the injection site accurately, we first reproduced the work of Yin et al. (2019) to obtain the 3D distribution of the MEPs in two commonly studied muscles, the gastrocnemius and the tibialis anterior (Figures 1A,B,D,E). Based on the lamellar distribution characteristics of MEPs, we determined the detailed injection approach for each muscle. For the gastrocnemius, we determined seven injection sites based on the “M” distribution, with a depth of 1.2–1.5 mm, and each site was injected with 500 nl CTB-647 (1 μg/μl), as shown in Figure 1C. For the tibialis anterior, we designed four injection sites with 500 nl at each site according to the “triangular pyramid” distribution of MEPs, of which the injection depth at the three sites (blue asterisk) was 0.6–0.8 mm and the injection depth of one site (yellow asterisk) was 1.3–1.5 mm (Figure 1F).

To compare with the injection approach based on the MEP region on the muscle surface (denoted as “2D intramuscular injection”), we obtained the MEP location on the gastrocnemius and the tibialis anterior surface using acetylcholinesterase histochemical staining (Mohan et al., 2015a), as shown in Figures 1G,I. For 2D injection, the gastrocnemius was injected on seven sites (each site with 500 nl), with a depth of 1.2–1.5 mm (Figure 1H), and the tibialis anterior was injected on four sites with 500 nl each at a depth of 0.6–0.8 mm (Figure 1J). The total injection volume was kept the same with the 3D injection. The injection details are concluded in Table 1.

**TABLE 1:** Details for 3D and 2D intramuscular injection strategies.

Injection strategy	Muscle	Injection sites	Injection depth (mm)	Volume of CTB (μl)	Volume/site (nl)
3D injection	Gastrocnemius	7	1.2–1.5	3.5	500
	Tibialis anterior	4	0.6–0.8/1.3–1.5^a^	2.0	500
2D injection	Gastrocnemius	7	1.2–1.5	3.5	500
	Tibialis anterior	4	0.6–0.8	2.0	500

*^a^Among the four sites for 3D injection of the tibialis anterior, one site was injected deeper than the other three, as shown in Figure 1F.*

### 3D Intramuscular Injection Approach Improves the Diffusion of CTB in Skeletal Muscle

Furthermore, to investigate whether 3D intramuscular injection enables the tracer to cover more MEP regions and further maximizes retrograde transport, we evaluated the quantification of the coverage between the diffusion region of the tracer and the MEP distribution after the intramuscular injections.

**FIGURE 2:**
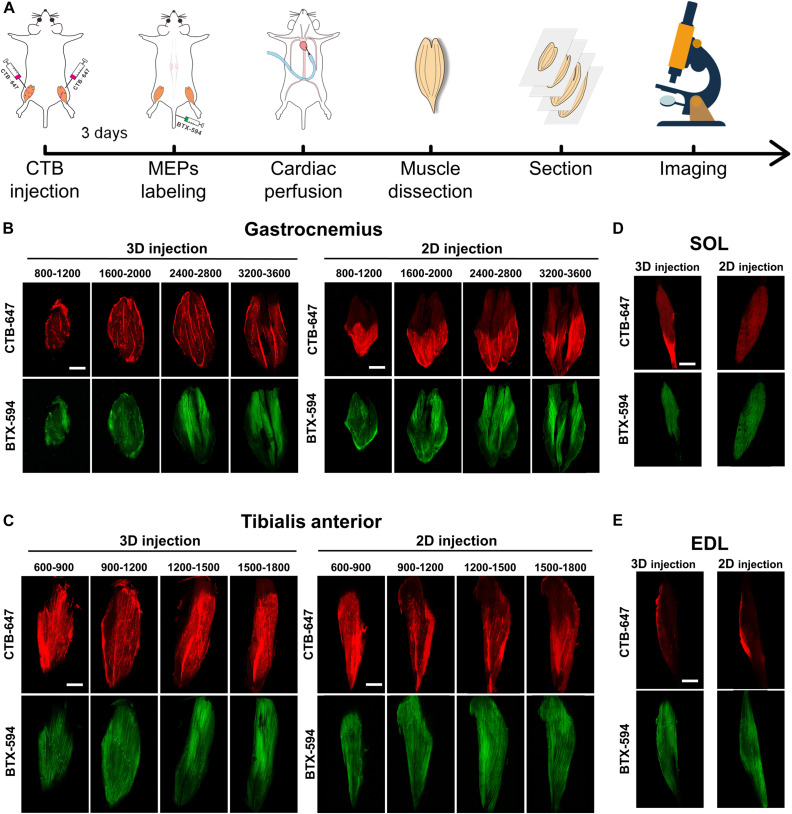
The cholera toxin subunit B (CTB) diffusion and the motor endplate (MEP) distribution in the muscle sections. **(A)** The entire experiment process of detecting the CTB and the MEPs in the muscle sections. **(B,C)** The distribution of the CTB and the MEPs at different depths (μm) of the gastrocnemius and tibialis anterior after 3D injection and 2D injection. **(D,E)** Images in the adjacent soleus and extensor digitorum longus. Each image is a maximum intensity projection of the image stacks (60-μm thickness). Scale bar: 2,000 μm.

Figure 2A shows the entire experiment process to detect the CTB tracer and the MEPs in the muscle section. At 3 days after the intramuscular injection of CTB-647, the mice were injected with α-BTX 594 to label the MEPs in the skeletal muscle (*n* = 4 for the gastrocnemius and tibialis anterior). After perfusion and dissection, the muscles were sectioned continuously in a horizontal direction and subsequently imaged by confocal microscopy. The distribution of the tracer and the MEPs in the gastrocnemius and the tibialis anterior after the intramuscular injection was demonstrated at different depths in Figures 2B,C, respectively. The results showed that the CTB diffusion by 3D injection is larger than that by 2D injection.

Moreover, considering the possibility of CTB leaking into the adjacent muscles, we imaged the SOL and EDL. The results in Figures 2D,E showed that only a little tracer was detected on the SOL and EDL surface resulting from the prevention of the fascia, which demonstrated the labeling specificity of motor neurons corresponding to the target muscles.

**FIGURE 3:**
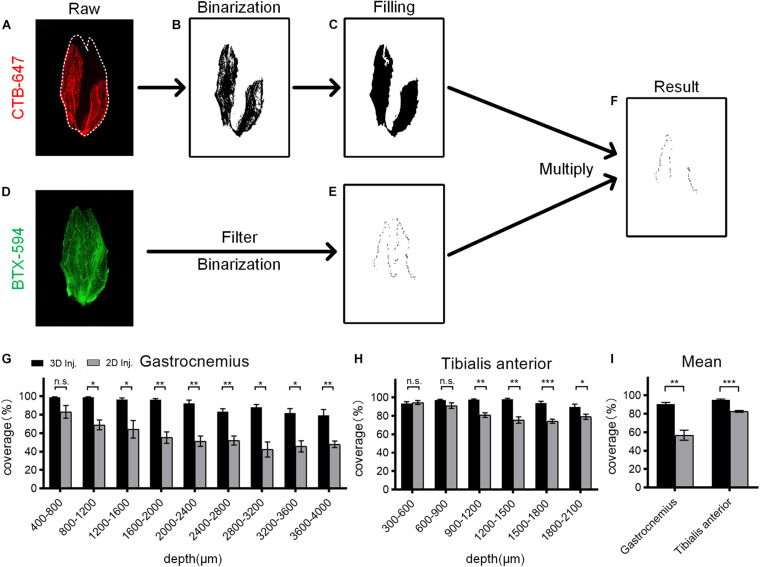
Quantitative analysis of the coverage ratio between the cholera toxin subunit B (CTB) region and the motor endplate (MEP) distribution. **(A)** Raw fluorescence image of the CTB in the muscle section. **(B)** Segmentation of the CTB region by image binarization. **(C)** The binary image was processed by filling holes resulting from the heterogeneity of fluorescence signal to determine the CTB region. **(D)** Raw fluorescence image of the MEP distribution on the muscle section. **(E)** Segmentation of MEP distribution by image filtering and binarization. **(F)** The result by multiplying the binary images of the CTB region and MEP distribution. **(G)** Comparison of the coverage at different depths of the gastrocnemius after the 3D and 2D injections (*n* = 4). **(H)** Comparison of the coverage at different depths of the tibialis anterior after the 3D and 2D injections (*n* = 4). **(I)** The mean coverage of the gastrocnemius and tibialis anterior after two injection strategies. Data are presented as mean ± SEM. The statistical significance (n.s. represents no significance, *p < 0.05, **p < 0.01, ***p < 0.001) in panels **(G–I)** was mainly assessed by independent-sample *t*-test. Some groups in panel **(G)** were assessed by the Mann–Whitney *U*-test or the separate variance estimation *t*-test due to the non-normal distribution or the heterogeneity of variance.

To quantitatively compare the CTB diffusion after two injection strategies, we developed a method to calculate the coverage ratio between the CTB diffusion region and the MEP distribution (Figures 3A–F). The raw fluorescence image was initially processed by image binarization to extract the signal area, and then the binary image was processed by the hole-filling algorithm to get an accurate segmentation of the CTB diffusion region. Then, for the segmentation of MEPs, raw fluorescence image was preprocessed by filtering to reduce the interference from muscle autofluorescence, followed by binarization. Due to differences in the fluorescence intensity of the background and signal in different images, the threshold for binarization of each image is manually adjusted to extract the signal region. Finally, the binary image of the MEP distribution was multiplied with the binary image of the CTB diffusion region. The coverage was defined as the ratio of the number of pixels of the MEP signal in the CTB region to the total number of pixels of the MEP signal. It should be noted that the binarization images in the whole process are inverted (Figures 3B,C,E,F) in order to make the extracted signal distribution easy to be observed.

For gastrocnemius, the 3D injection resulted in a significantly higher coverage ratio than the 2D injection at each depth, with an average increase of 33.91% (Figures 3G,I). Similarly, for tibialis anterior, the mean coverage ratio of the 3D injection was 12.46% higher than that of the 2D injection (Figures 3H,I). These results demonstrate that the 3D injection allows the tracers to reach more MEP regions and ensures a greater uptake of the tracers by the MEPs, thereby maximizing the retrograde transport.

### 3D Intramuscular Injection Approach Maximizes the Retrograde Labeling of Spinal Motor Neurons

**FIGURE 4:**
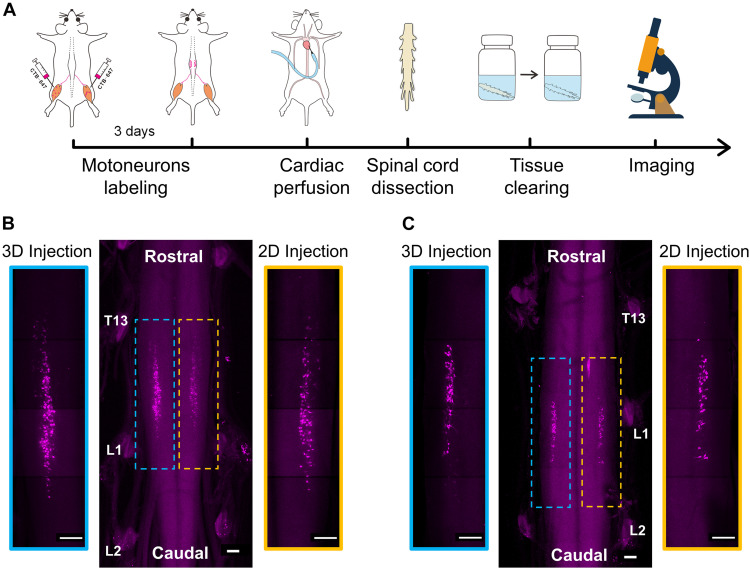
3D imaging of spinal motor neurons innervating the muscles after intramuscular injection. **(A)** The entire experimental process of 3D imaging for the spinal cord. **(B)** Maximum intensity projection (MIP) of the image stacks of labeled motor neurons corresponding to the gastrocnemius. The enlarged images indicated by blue and yellow dotted boxes showed the distribution of motor neurons labeled by the 3D and 2D injections, respectively. **(C)** MIP images of labeled motor neurons corresponding to the tibialis anterior after the 3D and 2D injections, respectively. Scale bar: 300 μm.

With the above-mentioned injection strategies, we injected the retrograde tracer into the muscles to label motor neurons in the spinal cord and obtained the motoneuron distribution by the combination of optical clearing and imaging (Figure 4A). Two injection strategies were administered to the muscles from the left and right limbs in the same mouse, respectively, to avoid the labeling differences resulting from individual differences. After the intramuscular injections, the mice were kept for 3 days to ensure the optimal retrograde transport of CTB. After cardiac perfusion and dissection, the spinal segments between T12 and L2 were dissected and cleared with FDISCO and imaged by confocal microscopy.

The results in Figures 4B,C show the distribution of motor neurons corresponding to the gastrocnemius and tibialis anterior, respectively. In general, the motor neurons were concentrated in a single, longitudinal pool in the spinal cord anterior horn. The motor neuron pool innervating the gastrocnemius was mainly located in the spinal segments between T13 and L1 nerve root (Figure 4B). The motor neuron pool innervating the tibialis anterior was shorter and located in the spinal segment near the L1 nerve root (Figure 4C). The qualitative analysis of labeled neurons on the left and right sides of the spinal cord indicated that the efficiency of the retrograde transport by the 3D injection strategy is higher than that of the 2D injection strategy.

**FIGURE 5:**
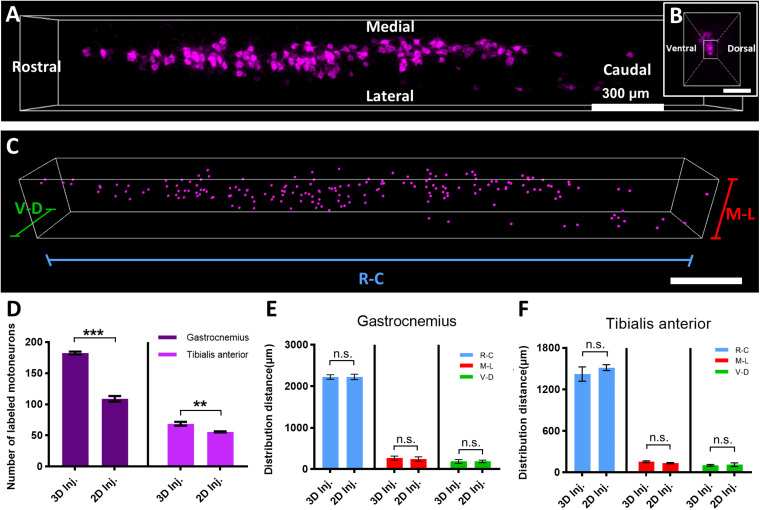
Quantitative comparison of the distribution characteristics of labeled motor neurons by the two injection strategies. **(A)** 3D reconstruction of labeled motor neurons in the spinal cord. **(B)** The cross-sectional view corresponding to panel **(A)**. **(C)** The quantification of distribution characteristics of motor neurons *via* the Spot and Vantage modules in Imaris. **(D)** Comparison of the neuron number of the gastrocnemius and tibialis anterior labeled by the two injection strategies (*n* = 4 for the gastrocnemius and tibialis anterior). **(E,F)** Comparison of the distribution range of motor neuron pools corresponding to the gastrocnemius and tibialis anterior labeled by the two injection strategies (*n* = 4 for the gastrocnemius and tibialis anterior). Data are presented as mean ± SEM. Statistical significance (n.s. represents no significance, **p < 0.01, ***p < 0.001) was assessed by independent-sample *t*-test.

To quantitatively compare the labeling efficiency by the two injection strategies, we reconstructed the 3D distributions of the motor neurons (Figures 5A,B) and analyzed their characteristics (Figure 5C) by Imaris. First, we counted the number of labeled motor neurons. The results in Figure 5D showed that, for the gastrocnemius, the number of neurons labeled by the 3D injection is 183 ± 2, while the number of neurons labeled by the 2D injection is 109 ± 4. The efficiency of the retrograde transport based on the 3D injection was improved by 67.89%. Similarly, for the tibialis anterior, the efficiency of the 3D injection was improved by 23.21% compared to the 2D injection. These quantitative results indicated that the injection method based on the 3D distribution of MEPs could enhance the efficiency of the retrograde transport compared to that based on the 2D distribution of MEPs on the muscle surface, and the larger the muscle volume, the more significant the improvement in the efficiency might be.

Then, we compared the distribution range of motor neuron pools targeting the gastrocnemius and tibialis anterior (Figures 5E,F) by the two injection strategies. For the 3D injection approach, the labeled motor neuron pool innervating the gastrocnemius distributed mainly from the rostral to the caudal direction (R–C) with a range of 2,225.47 ± 23.78 μm, medial–lateral (M–L) with a range of 265.52 ± 20.76 μm, and ventral–dorsal (V–D) with a range of 188.56 ± 18.88 μm, while for the 2D injection approach, the distribution ranges of the pool in the three directions are 2,225.50 ± 28.91, 243.75 ± 22.46, and 188.80 ± 13.00 μm, respectively. The statistical results showed that, for gastrocnemius, there was no significant difference in the distribution range of the neuron pools labeled by the 3D and 2D injection approaches. The distribution ranges of the motor neuron pool innervating the tibialis anterior demonstrated a smaller range and also showed no significant difference between the two injection strategies.

## Discussion

In this study, a new intramuscular injection strategy based on the 3D distribution of the MEPs to label spinal motoneurons was proposed. This 3D intramuscular injection strategy allows the retrograde tracer to cover the entire MEP region. It maximizes the retrograde transport to label spinal motor neurons to perfect the muscle–motoneuron topographical organization.

Understanding the anatomical relationship between the muscles and the motor neurons enables the delivery of therapeutic gene vectors to specific spinal cord segments by intramuscular injection in gene therapy. Retrograde tracing techniques by injecting retrograde tracers into muscles have been widely applied. The labeling efficiency depends on whether the tracer is located near the MEP region. Thus, the researchers tried to locate the MEP region and targeted the region with tracers to improve the labeling efficiency. Morris et al. located the MEP regions on the surface of multiple muscles, injected fluoro-gold along these regions, and systematically described the detailed distribution characteristics of the motor neurons (Tosolini and Morris, 2012; Tosolini et al., 2013; Mohan et al., 2014, 2015b). However, the MEPs are not evenly distributed in the skeletal muscles (Nandedkar et al., 1988; Prodanov et al., 2005; Navallas and Stålberg, 2009), and each skeletal muscle has distinctive distribution characteristics (Yin et al., 2019). Injecting along the MEP region on the muscle surface could improve the labeling efficiency compared to a “blind injection” but is still limited, especially for large muscles with extensive and complex MEP distribution, such as the gastrocnemius—for example, Mohan et al. (2014) demonstrated that the number of motor neurons targeting the gastrocnemius was even less than that targeting the tibialis anterior. In the study of Han et al. (2019), to improve the labeling efficiency, the tracers were injected until the muscles were completely colored, ensuring that the whole muscles were filled with the tracers, but the availability of this approach is still limited for the large muscles because the color change in the appearance of the muscle could not accurately reflect the diffusion of the tracer inside the muscle. Moreover, “filling injection” not only requires more tracers and cost but also may cause damage to the muscle itself. In short, the current injection strategies are challenging to reach the maximum labeling efficiency. Benefiting from the development of tissue clearing technique and optical imaging in recent years (Zhu et al., 2013; Richardson and Lichtman, 2015; Ueda et al., 2020), the acquisition of the 3D distribution of MEPs in whole-mount skeletal muscles provides new guidance for intramuscular injection.

The quantitative analysis of retrogradely labeled motor neurons based on the two injection strategies proved that the injection strategy targeting the 3D distribution of MEPs (3D injection) is more effective to improve the retrograde transport, especially for the large muscle. It is worth noting that the number of motor neurons innervating the gastrocnemius labeled by the 2D injection in this study was twice than that previously reported (Mohan et al., 2014; Han et al., 2019). There were no CTB signals in the soleus attached to the gastrocnemius (Figure 2D), suggesting that the greater number of neurons in this work is not due to the tracer leaking into the non-target muscles.

For the tibialis anterior, although the CTB covered 94.83% of the MEP region after the 3D injection in Figure 3I, the number of labeled neurons by the 3D injection was much lower than that in the research of Morris’s group (Mohan et al., 2014). It is speculated that the difference in the number of labeled neurons involves two possible reasons. On the one hand, the traditional histological methods were used to count the number of motor neurons, including sectioning, imaging, and manual counting, leading to the potential double-counting of motor neurons in adjacent sections (Žygelytė et al., 2016). On the other hand, the EDL, a muscle embedded in the side of the tibialis anterior, is always injected at the same time by mistake when the target is the tibialis anterior in practice, leading to an excessive counting of labeled motor neurons. We used the microscopic tweezer to enlarge the space between the tibialis anterior muscle and the EDL prior to injection to avoid spurious labeling, and the imaging result of the EDL in Figure 2E shows that the above-mentioned operation is available and our labeling results are accurate.

The injection strategy based on the 3D distribution of MEPs can significantly improve the retrograde transport, and the larger the skeletal muscles, the more significant the improvement in labeling efficiency. In other words, the larger the skeletal muscles, the more necessary the 3D injection. Thus, the 3D injection shows positive implications for future studies and clinical therapeutics. On the one hand, in combination with tissue clearing, retrograde tracing by 3D injection, and whole-mount analysis, the anatomical relationship between the skeletal muscles and the motor neurons will be updated and refined in future research. On the other hand, the 3D intramuscular injection is expected to provide a tool to explore novel treatment strategies for spinal cord injury and diseases—for instance, the results of preclinical trials *via* the intramuscular injection of viral vectors containing therapeutic genes were always unsatisfactory, in part because it was difficult for the therapeutic vectors to reach the targeted neurons effectively by the current injection methods. In addition, MEP is not only an access for the retrograde transport of tracers or viral vectors but also a therapeutic target itself. Clinically, botulinum toxin A provides a local tone reduction to reduce spasticity by blocking neurotransmission at the MEPs (Delnooz et al., 2014), but the effect of botulinum toxin A is dose dependent, and in the case of suboptimal injection strategies, the excessive dose can result in systemic side effects or weakness of adjacent muscles and economic waste (Van Campenhout and Molenaers, 2011; Yang et al., 2016). The 3D injection strategy proposed in this work would potentially achieve optimal therapeutic outcomes with a minimal effective dose.

## Data Availability Statement

The raw data supporting the conclusions of this article will be made available by the authors, without undue reservation.

## Ethics Statement

The animal study was reviewed and approved by Institutional Animal Ethics Committee of Huazhong University of Science and Technology.

## Author Contributions

JX and TY conceived and designed the study and wrote the manuscript. AX and YL performed intramuscular injections. AX and JX performed tissue clearing and imaging. JX, AX, and ZL performed image processing and analysis. TY and YY supervised the project and revised the manuscript. JZ and DZ gave valuable comments and suggestions for this study. All authors read and approved the final manuscript.

## Conflict of Interest

The authors declare that the research was conducted in the absence of any commercial or financial relationships that could be construed as a potential conflict of interest.

## Publisher’s Note

All claims expressed in this article are solely those of the authors and do not necessarily represent those of their affiliated organizations, or those of the publisher, the editors and the reviewers. Any product that may be evaluated in this article, or claim that may be made by its manufacturer, is not guaranteed or endorsed by the publisher.
